# The Lysosomal Diseases Testing Laboratory: A review of the past 47 years

**DOI:** 10.1002/jmd2.12117

**Published:** 2020-04-04

**Authors:** David A. Wenger, Paola Luzi

**Affiliations:** ^1^ Department of Neurology Sidney Kimmel College of Medicine at Thomas Jefferson University Philadelphia Pennsylvania USA

**Keywords:** genetic complementation, GM1 gangliosidosis, Krabbe disease, lysosomal diseases, metachromatic leukodystrophy, newborn screening, storage diseases

## Abstract

Lysosomal disorders are diseases that involve mutations in genes responsible for the coding of lysosomal enzymes, transport proteins, activator proteins and protein processing enzymes. These defects lead to the storage of specific metabolites within lysosomes resulting in a great variety of clinical features depending on the tissues with the storage, the storage products and the extent of the storage. The methods for rapidly diagnosing patients started in the late 1960's when the enzyme defects were identified eliminating the need for tissue biopsies. The first requests for diagnostic help in this laboratory came in 1973. In that year, patients with Krabbe disease and Niemann‐Pick type A were diagnosed. Since that time samples from about 62 000 individuals have been received for diagnostic studies, and 4900 diagnoses have been made. The largest number of diagnosed individuals had metachromatic leukodystrophy and Krabbe disease because of our research interest in leukodystrophies. A number of new disorders were identified and the primary defects in other disorders were clarified. With new methods for diagnosis, including newborn screening, molecular analysis, microarrays, there is still a need for biochemical confirmation before treatment is considered. With new treatments, including gene therapy, stem cell transplantation, enzyme replacement used alone or in combination becoming more available, the need for rapid, accurate diagnosis is critical.

**SYNOPSIS**

After 47 years the laboratory has diagnosed 4900 patients with a lysosomal storage disease and has characterized some new diseases.

1

In this manuscript we review the history of the Lysosomal Diseases Testing Laboratory (LDTL) from the initial requests for testing in 1973 to the present. In addition to providing information on the number of patients with each disorder diagnosed, we will highlight some of the more interesting cases that opened new thinking about this group of genetic disorders. In 1971, David A. Wenger was offered a position as an Assistant Professor of Pediatrics at the University of Colorado Medical Center. There was some initial funding from the B. F. Stolinsky family because of a niece with Gaucher disease. The only stipulation was for him to do research on lysosomal disorders. He was recruited from the laboratory of Dr. John S. O'Brien, who first identified the deficiency of hexosaminidase A activity in patients with Tay‐Sachs disease, where he was a postdoctoral fellow. About that time the biochemical defects were being identified opening the way to rapid and simpler diagnostic tests which eliminated the need for brain or other organ biopsies. The measurement of enzymes activities in blood samples or cultured skin fibroblasts became the standard for diagnostic testing. Another advancement that improved the sensitivity of the diagnostic testing was the increasing commercial availability of fluorogenic substrates replacing colorimetric and natural substrates. At that time, the genes for the lysosomal enzymes and proteins had not been cloned but that would begin within the next 15 years opening additional options for diagnosis, for genotype‐phenotype correlations and eventually for treatment.

Initial research projects in the laboratory included studies on Krabbe disease, Niemann‐Pick types A, B, and C, and Gaucher disease. Mainly these studies involved examination of autopsy tissues from patients who died with these disorders to look for biochemical changes that could lead to a better understanding of the pathogenesis of the diseases. In 1970, Malone^1^ and Suzuki and Suzuki^2^ found that patients with Krabbe disease were deficient in the beta‐galactosidase activity that catalyzed the hydrolysis of galactose from galactosylceramide, a key sphingolipid in myelin. Patients with GM1 gangliosidosis were deficient in another beta‐galactosidase that catalyzed the hydrolysis of the terminal galactose from GM1 ganglioside, GA1, keratan sulfate as well as glycoproteins, but not galactosylceramide.

In 1973 a child neurologist in the Department of Pediatrics saw a 3‐month‐old child who was developmentally delayed, extremely irritable, and stiff. She suspected a disorder like Krabbe disease or Tay‐Sachs disease and asked if the laboratory could help make a diagnosis. The assays for these enzymes were already in use, but had not been used for diagnostic studies. Blood was taken from laboratory personnel and the patient, and leukocytes were isolated using dextran according to a published procedure.^3^ The results showed near zero galactocerebrosidase (GALC) activity and normal activity for other lysosomal enzymes confirming the diagnosis of Krabbe disease. The same year there was a request for testing of a 6‐month‐old child with developmental delay, an enlarged liver, and bilateral macular cherry‐red spots. Very low acid sphingomyelinase activity was measured, and a diagnosis of Niemann‐Pick type A was made. In 1973, the laboratory had less than ten requests for diagnostic help. Additional request for testing started to come from centers outside of Colorado. After determining that enzymes could be measured in isolated leukocytes and plasma from unclotted blood received 1 or 2 days after collection, word went out that the laboratory would be able to help with diagnostic testing in patients from around the country. The selection of tests to be assayed on each patient is based on suggestions from the sending physician and our suggestions from the clinical information supplied with the sample. These diseases are not common and many physicians may only see one or two patients during their entire medical career. By receiving clinical information with each sample, we could identify some key findings that might suggest testing that would not be obvious to the physician (Table 1). The increase in samples received continued even when LDTL moved from Denver to Jefferson Medical College in Philadelphia in 1986.

**Table 1:** Key features that determine which tests are performed

1. Developmental delay and/or regression of previous learned skills, ataxia, spasticity, hypotonia, behavioral changes, white matter changes on MRI, seizure, and muscle weakness (without presence of coarse facial features, short stature, organomegaly, etc.)
– GM1 and GM2 gangliosidoses, metachromatic leukodystrophy, Krabbe disease, β‐mannosidosis, α‐fucosidosis, Pompe disease.
2. Coarse facial features, dysostosis multiplex or short stature with or without developmental delay, organomegaly, eye changes etc.
– GM1 and GM2 gangliosidoses, MPS I, IIIB, VI and VII, α‐fucosidosis, α and β‐mannosidosis, ML II and III, sialidosis, galactosialidosis, sialuria, sialic acid storage disease, and multiple sulfatase deficiency.
3. Organomegaly with or without developmental delay
– Gaucher disease, Niemann‐Pick types A, B and C, Wolman disease, cholesteryl ester storage disease.
4. Eye findings
(A) Macular cherry‐red spots
– GM1 and GM2 gangliosidoses, Niemann‐Pick types A, B and C, sialidosis, galactosialidosis, Krabbe disease.
(B) Corneal clouding
– GM1 gangliosidosis, MPS I, VI and VII, α‐fucosidosis, α‐mannosidosis, β‐mannosidosis, ML II and III, sialidosis, galactosialidosis, Fabry disease, and multiple sulfatase deficiency.
(C) Miscellaneous eye finding
– Tortuosity of conjunctival vessels (Fabry disease, α‐fucosidosis)
– Vertical supranuclear ophthalmoplegia (Niemann‐Pick type C)
5. Miscellaneous features
(A) Angiokeratomas
– Fabry disease, α‐fucosidosis, GM1 gangliosidosis, sialidosis, galactosialidosis, β‐mannosidosis.
(B) Nonimmune fetal hydrops
– GM1 gangliosidosis, Gaucher disease, Niemann‐Pick types A, MPS VII, Sialidosis, galactosialidosis, sialic acid storage disease, MPS I, ML II.
(C) Light pigmented skin
– Sialic acid storage disease

At this time samples from about 62 000 individuals have been received by this laboratory for testing. This has resulted in about 4900 definitive diagnoses (a “hit” rate of about 8%; Table 2). It should be understood that this laboratory never tested for all of the lysosomal disorders. Some of the mucopolysaccharidoses (MPS), including MPS II, MPS III A, C, and D, MPS IVA and other diseases were never tested for and testing was stopped for other disorders due to a lack of requests (eg, Farber disease). Testing for some disorders, like Pompe disease and Niemann‐Pick type C, came later. A decision was made to not charge by the test but to charge a flat rate for all tests performed. This allowed us to perform all tests indicated by the information provided to try to arrive at a diagnosis, even if the probability of a diagnosis was slim. This philosophy also resulted in some unexpected diagnoses on patients with atypical presentations. That is the advantage of having clinical information on thousands of samples received here. Not all tests available in the laboratory are run on every individual, however, we are liberal in our test selection knowing that not all patients have a “classical” presentation. We feel it is not necessary to test for certain disorders if certain key features are not mentioned.

**Table 2:** Number of patients diagnosed in the Lysosomal Diseases Testing Laboratory since 1973

Disease and number diagnosed	Disease and number diagnosed
GM1 gangliosidosis (+MPS IVB) 252	Mucolipidosis II and III 246
GM2 gangliosidosis (all types^a^) 451	α‐mannosidosis 49
Metachromatic leukodystrophies 909	β‐mannosidosis 6
Krabbe disease 771	Fucosidosis 49
Gaucher disease 558	MPS IIIB 70
Niemann‐Pick disease (all types^b^) 583	MPS VII 40
Fabry disease 124	Sialidosis, Sialuria, SASD^c^ 82
Wolman & CESD^d^ 50	Galactosialidosis 40
MPS I (H, HS, S^e^) 479	Multiple sulfatase deficiency 27
MPS VI 86	Farber disease 8
Pompe disease 16	

*Note*: Total = 4896.

a Includes Tay‐Sachs disease, Sandhoff disease, and GM2 activator protein deficiency.

b Includes Niemann‐Pick type A, B, and C.

c Sialic acid storage disease.

d Cholesterol ester storage disease.

e Includes Hurler, Hurler‐Scheie, and Scheie syndromes.

Looking at Table 2 it may be surprising that the diseases with the highest number of diagnoses are metachromatic leukodystrophy (MLD) and Krabbe disease. However, this reflects a number of factors including the research interests of the laboratory in leukodystrophies and the availability of radiolabeled galactosylceramide for the diagnosis of Krabbe disease. In addition, the commercial availability of synthetic substrates increased the number of laboratories that can perform the tests for certain lysosomal disorders. It is assumed a larger number of patients would be diagnosed with Gaucher disease and MPS I as was found in Brazil by Giugliani et al.^4^ Samples come to the LDTL from all over the United States which now has a very diverse population compared to 35 to 45 years ago when samples tended to come more from a northern European population (based on the surnames of the individuals). From the data on the Table 2 and the reasons provided, it is not possible to assign a frequency for each of these diseases.

All lysosomal disorders include patients with a wide range of ages of onset and clinical course, but for the purpose of this publication this was not taken into account. The number of diagnosed patients with the same disease designation are listed together. Patients with mutations in the same gene can have a very different clinical picture. For example, patients with GM1 gangliosidosis and MPS IVB have mutations in the acid beta‐galactosidase gene. Different mutations can change the enzyme's ability to hydrolyze the terminal beta‐linked galactose moiety from different substrates resulting in different storage products in different organs. In 1970, Dawson and Stein^5^ described a patient with a neurovisceral disease, evidence for storage of lactosylceramide and other lipids in liver and low lactosylceramide beta‐galactosidase activity. It was called lactosylceramidosis. Studies using different assay conditions did not show a defect in hydrolyzing the beta‐linked galactose from lactosylceramide,^6^ and additional studies by this laboratory showed that the patient had Niemann‐Pick type C.

In addition, there are patients who are phenotypically very similar and share the same pathological features but have mutations in different genes. This is true for the 450 patients diagnosed with GM2 gangliosidosis. Some of these patients have Tay‐Sachs disease (deficiency of hexosaminidase A, Hex A), some have Sandhoff disease (deficiency of hexosaminidases A and B, Hex A and B) and some have normal measured hexosaminidase activity (deficiency of GM2 activator protein). The differential diagnosis of GM2 gangliosidoses can be simple but requires the ability to separately measure Hex A and Hex B activities using differences in heat stabilities of Hex A and B and by using a sulfated synthetic substrate that almost exclusively measures Hex A activity.^7^ There are patients with GM2 gangliosidosis who have mutations in the α‐chain of Hex A that will only be diagnosed using the sulfated substrate and could be missed using the heat denaturation method. It is called the B1 variant of GM2 gangliosidosis. When we first published on such a patient in 1983 we called the disease GM2 gangliosidosis Hex A^m^B variant to indicate that the α‐chain of Hex A has a mutation missed by the heat denaturation method.^8^ Patients who clinically resemble GM2 gangliosidosis but who have normal Hex A and B activity may have a deficiency of GM2 activator protein required for bringing GM2 ganglioside together with Hex A. The diagnosis was made in six patients by looking at the increase in GM2 ganglioside in cerebrospinal fluid using a method developed in this laboratory.^9^

The laboratory also made some discoveries related to the recognition of new lysosomal diseases and the clarification of the cause of previously described disorders. By 1980, several articles were published describing patients with low acid beta‐galactosidase activity but varying clinical presentations.^10^, ^11^, ^12^, ^13^ Also, Hans Galjaard and coworkers reported that when cultured cells from certain patients with “GM1 gangliosidosis” were grown together and chemically fused there was an increase in acid beta‐galactosidase activity showing genetic complementation.^14^ This laboratory had diagnosed a juvenile patient with low (about 10% of normal) beta‐galactosidase activity in leukocytes and cultured skin fibroblasts. The mother did not have carrier levels of beta‐galactosidase activity. Seeing clinical features similar to patients with sialidase deficiency, the activity of sialidase was measured in the cultured fibroblasts of the patient, and very low activity was measured.^15^ Therefore this patient had a combined deficiency of beta‐galactosidase and sialidase activities. It came to be called galactosialidosis. Alessandra d'Azzo and colleagues discovered that patients with galactosialidosis had reduced levels of a “protective protein,” later found to be cathepsin A needed for lysosomal localization and activation of both acid beta‐galactosidase and sialidase.^16^

In 1981, Jones and coworkers described a goat with very low β‐mannosidase activity.^17^ This enzyme is needed for the lysosomal hydrolysis of β‐linked mannose residues found primarily in glycoproteins. At that time no human patients with β‐mannosidase deficiency had been described. Therefore, the laboratory developed an assay for β‐mannosidase activity and tested every sample for this enzyme along with other lysosomal enzymes. Within 6 months of starting to test for this enzyme a patient with very low β‐mannosidase activity in leukocytes and plasma was identified.^18^ He excreted the expected product, mannose‐β‐N‐acetyglucosamine, in his urine. The unrelated parents had about half normal β‐mannosidase activity as expected for carriers of an autosomal recessive disease. However, additional testing of the urine showed that he was also excreting excess glycosaminoglycans, mainly heparan sulfate. Further testing showed that he was also deficient in heparan *N*‐sulfatase activity as found in patients with Sanfilippo type A (MPS IIIA). This is not the only time a patient was found to have two different lysosomal disorders. However, it is extremely unusual for this to happen with such a very rare disease such as β‐mannosidosis, where only five additional patients have been diagnosed in the LDTL since 1986.

The gene for arylsulfatase A (ARSA) was cloned in 1990,^19^ and since then over 200 mutations (hgmd.cf.ac.uk) have been identified in patients with MLD. While this disease is pan‐ethnic, this laboratory diagnosed a number of late infantile patients in the Navajo Indian and Yupik Eskimo populations. An MD‐PhD student in the laboratory, Nuria Pastor‐Soler, initially found the mutation causing MLD in Navajo children. This mutation was homozygous in all the affected children and heterozygous in the parents.^20^ She then looked for the mutation in the Eskimo children, not expecting it to be the same one found in the Navajos. However, they have the same mutation as the Navajo that has never been found in any other population in the world.^21^ Our studies show that the Navajo and Yupik Eskimo populations interacted exchanging genetic information.

Diagnosing patients with Gaucher disease became more important after the development of enzyme replacement therapy in 1990.^22^ This is a disease with a wide age range of patients from newborns to the elderly. The clinical features in non‐neuronopathic Gaucher disease (type 1), even in siblings with the same genotype, can be quite variable. Many years can separate the onset of clinical features. However, in almost all cases, if one sibling has the non‐neuronopathic form, the other siblings will also, and conversely the same is true of the acute neuronopathic form (type 2). However, in one family we identified two siblings who died at eight months and one year with type 2 Gaucher disease and a 25‐year‐old full sibling who was mildly affected with type 1 Gaucher disease.^23^ At that time the mother and father were clinically normal. Enzymatic testing showed that the father had a carrier level of glucocerebrosidase activity, and the mother had glucocerebrosidase activity in the affected range for Gaucher disease. Later studies showed that the father carried a severe mutation (L444P) and the mother was a compound heterozygote with a severe (L444P) and a mild mutation (N370S). The two children with acute neuronopathic Gaucher disease inherited two copies of the severe mutation and the mildly affected son inherited the father's severe mutation and the mother's mild mutation. The mother was later found to have moderate splenomegaly. The son and his mother have an identical genotype (N370S/L444P) with considerable variability in clinical presentation.^24^

The methods used for biochemical testing in this laboratory have not changed much in 47 years.^25^ However, once the genes for the lysosomal proteins were cloned, methods for detecting mutations were developed. Rapid methods for isolating DNA from very small tissue samples, even dried blood spots (DBS), and development of polymerase chain reaction have changed the options available for testing patients. Also, the price for mutation‐based testing has dropped dramatically in the last five years. However, the identification of one or two mutations in a gene does not automatically make the diagnosis in an individual. Many “mutation positive” cases require follow‐up testing using biochemical methods to confirm that what was found molecularly is actually disease causing. Also, it is essential to obtain a diagnosis as quickly as possible before significant pathological changes occur especially in the nervous systems. This has resulted in newborn screening (NBS) for a number of lysosomal disorders.^26^, ^27^ The diseases tested for and methods used vary from state to state, however most rely on enzymatic testing using DBS and tandem mass spectroscopy. While these methods are rapid and specific, they are screening tests, not diagnostic tests. Samples from infants who test positive in a NBS test arrive in this laboratory for confirmatory testing. Most of these screen‐positive infants will have additional testing including measurement of biomarkers, mutation analysis, and neurodiagnostic studies. As some treatments are expensive and not without risks, the diagnosis must be conclusive.

Another function of the LDTL is to measure the activity of enzymes in cord blood samples that will be used for hematopoietic stem cell transplantation. Also, once the transplantation has taken place the treated patients are followed closely for years, and blood samples are sent to the LDTL to measure the enzymatic activity to confirm that activity from the donor cells is still being maintained. Samples still arrive in the laboratory from individuals transplanted more than 20 years ago.

In addition to the patient testing by the LDTL there is also a research arm that has resulted in the cloning of several genes that are currently being used for diagnostic purposes and gene therapy. Studies on patients with features suggestive of MLD, but with normal arylsulfatase A activity resulted in the confirmation that these patients were deficient in a specific sphingolipid activator protein required for the enzymatic hydrolysis of sulfatide.^28^ The antibodies produced in this laboratory were used to show the deficiency of this activator protein in cultured cells from the patients and to clone the activator protein gene, now called prosaposin.^29^, ^30^ That lead to the identification of mutations in the prosaposin gene.^31^, ^32^

The earliest research efforts were to purify GALC, the enzyme deficient in patients with Krabbe disease. After nearly 20 years of research the GALC protein was purified,^33^ and amino acid sequence information lead to the cloning of the cDNA and gene.^34^, ^35^ Over 250 disease‐causing mutations in the GALC gene have now been identified (hgmd.cf.ac.uk), and this information is helpful in predicting the type of Krabbe disease an individual could have when identified in a NBS test.^27^ Also, published studies show that injection of GALC cDNA in an AAV viral vector into animal models of Krabbe disease along with bone marrow transplantation can correct the pathology of the disease and greatly extend the lives of treated animals.^36^ This treatment is moving toward a clinical trial in human patients. Therefore, the LDTL has served a useful function for 47 years and continues to provide information critical to the diagnosis and treatment of lysosomal disorders.

## CONFLICT OF INTEREST

The authors declare no potential conflict of interest.

## ETHICAL STATEMENT

All procedure followed were in accordance with the ethical standards of the responsible committee on human experimentation (institutional and national) and with the Helsinki Declaration of 1975, as revised on 2000

## PATIENT CONSENT STATEMENT

This article does not contain any studies with animal subjects performed by any of the authors.
